# Bioanalytical HPLC-UV Determination of Dopamine in Plasma and Mouse Brain Homogenate with Greenness, Whiteness, and Blueness Assessment

**DOI:** 10.3390/molecules31132255

**Published:** 2026-06-26

**Authors:** Miglena Smerikarova, Stanislav Bozhanov, Jana Tchekalarova, Petja Ivanova, Violina T. Angelova, Vania Maslarska

**Affiliations:** 1 Department of Chemistry, Faculty of Pharmacy, Medical University-Sofia, Dunav Str. no. 2, 1000 Sofia, Bulgaria; 2 Institute of Neurobiology, Bulgarian Academy of Sciences, 1431 Sofia, Bulgaria

**Keywords:** dopamine, plasma, brain, bioanalysis, greenness

## Abstract

Dopamine dysregulation is connected to several neurological disorders, including Parkinson’s disease, Huntington’s disease, and addiction. A new, precise, accurate, and specific reversed-phase high-performance liquid chromatographic method was developed for dopamine determination in different biological media (human/mouse plasma and mouse brain homogenate). The chromatographic assay was performed using Avantor ACE^®^ RP-18 (250 × 4.6 mm, 5 µm) column equipped with a suitable ODS pre-column. The temperature was ambient, and the mobile phase was composed of 10 mM potassium dihydrogen phosphate buffer (pH = 3) with 0.25 g/L sodium octanesulfonate, methanol, and acetonitrile at a volume-to-volume ratio of 75:20:5. Isocratic elution mode, flow rate 1.0 mL/min, and ultraviolet detection (280 nm) were applied. The procedure was validated for linearity, and all calibration curves were linear over the selected range with determination coefficients greater than 0.998. Intraday repeatability, expressed as the coefficient of variation, did not exceed 4.88% for the plasma and 3.32% for the mouse brain homogenate samples across all tested concentration levels. The proposed chromatographic method was evaluated in terms of greenness, whiteness, and blueness using three ecological metrics (the Analytical Greenness software, White Analytical Chemistry model, and Blue Applicability Grade Index). The optimized procedure was proven to be suitable for implementation in the routine analytical practice.

## 1. Introduction

**Figure 1:**
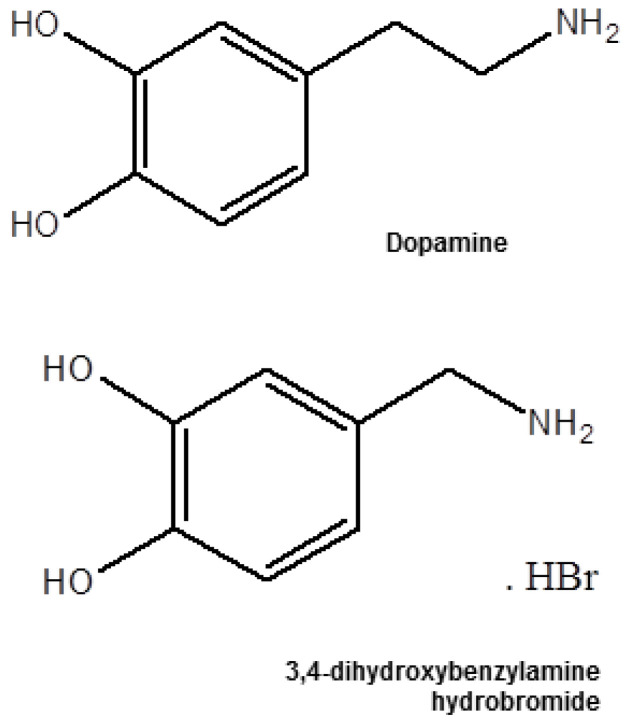
Chemical structures of DPM and internal standard.

The class of molecules, known as catecholamines, is primarily synthesized from the amino acid tyrosine and acts as neurotransmitters and hormones in various body regions [1]. These chemical substances include dopamine (DPM), norepinephrine (noradrenaline), and epinephrine (adrenaline) [2]. DPM (3-hydroxytyramine, Figure 1), produced in the brain’s substantia nigra and ventral tegmental area, serves multiple roles in the central nervous system, influencing mood, motivation, motor control, and gastrointestinal motility. It also acts as a vasodilator in the kidneys, regulating blood flow and sodium excretion, and modulating hormone release [1]. In connection with its physiological actions, DPM binds to specific membrane receptors of target cells, a part of the G protein-coupled receptor superfamily. Five subtypes of DPM receptors (D1, D2, D3, D4, and D5) have been identified, among which D1 and D2 are predominant in the brain. These metabotropic receptors initiate second messenger cascades that affect various cell signaling pathways [3]. DPM receptors are also presented in peripheral tissues, including blood vessels, kidneys, heart, retina, and adrenal glands, where they play key roles in controlling catecholamine release and the renin-angiotensin system [2].

Due to the crucial functions of DPM signaling, dysregulation is connected to several neurological disorders, including Parkinson’s disease (PD), Huntington’s disease, schizophrenia, attention deficit/hyperactivity disorder, and addiction. The progressive degeneration of dopaminergic neurons reduces DPM content in the substantia nigra and results in striatal DPM deficiency, which triggers the onset of PD clinical symptoms such as tremor, postural instability, bradykinesia, and muscle rigidity [4]. In the light of dopamine’s complex influence, a range of pharmacological treatments targeting DPM receptors, both agonists and antagonists, has been developed and used clinically to improve the symptoms of different conditions such as PD, hyperprolactinemia, schizophrenia, bipolar depression, and others [2,5,6]. Therefore, it is very important to develop precise, accurate, and specific methods for the quantification of DPM in different biological samples to help improve diagnosis and treatment [7,8]. Several bioanalytical methods have been developed to detect and quantify DPM in pharmaceutical and biological samples [9,10,11,12,13,14,15,16,17,18,19,20,21,22,23,24,25,26,27,28,29,30,31,32,33]. Among them, chromatographic techniques [9,10,11,12,13,14,15,16,17,18,19,20,21,22,23,24], including high-performance liquid chromatography (HPLC) [9,10,11,12,13,14,15,16,17,18,19,20,21], microdialysis [25,26], and capillary electrophoresis [27], have been widely used due to their high sensitivity, specificity, and resolution. Electrochemical [28,29,30], spectrophotometric [31], fluorimetric [32], and voltammetric [33] analysis were also applied. The literature survey showed that HPLC is one of the most often used techniques for the bioanalysis of DPM, offering high selectivity and the ability to separate complex mixtures of biological compounds. Reverse-phase HPLC (RP-HPLC) is the method of choice for DPM analysis, using an ultraviolet (UV) [9,10,11,12], fluorescence [13,14,15], mass [16,17,18,19], electrochemical [20], or amperometric [21] detector. RP-HPLC has been used extensively to monitor DPM levels in brain tissues, blood plasma, and urine. The method’s high resolution enables the detection of DPM and its metabolites with minimal interference from other catecholamines [13,18,19,20,21,23,24,27]. Moreover, HPLC is a highly effective tool in DPM kinetics monitoring, allowing for real-time measurements of patients’ plasma/tissue concentrations and providing insights about its role in various neurological disorders, including PD and addiction [13,18,21,22]. In the last five years, significant advancements in chromatographic techniques have improved the detection limits, speed, and specificity of DPM analysis in biological samples [34,35].

A simple isocratic reversed-phase HPLC-UV method for the determination of DPM was developed and validated in the present study. The procedure effectively estimated the target analyte in different biological matrices and was proven to be suitable for implementation in the routine analytical practice. Additionally, four quality assessment tools were used to analyze the method’s greenness profile and applicability—the Analytical Greenness software v0.5 beta (AGREE) [36], the Blue Applicability Grade Index software (BAGI, https://bagi-index.anvil.app/, accessed on 24 May 2026) [37], the Red Analytical Performance Index software v.0.92 (RAPI) [38], and White Analytical Chemistry tool (WAC) [39].

## 2. Results and Discussion

### 2.1. Optimization of Chromatographic Conditions and Experimental Parameters

The development and validation of the analytical procedure involved a comprehensive optimization of chromatographic conditions, including the selection of the stationary phase, column dimensions, mobile phase composition, flow rate, and operating temperature. A series of systematic experiments was therefore performed. Reverse-phase columns with octadecylsilane (C18) and octylsilane (C8) stationary phases, differing in length (150 and 250 mm) and internal diameter (4.0 and 4.6 mm), were comparatively assessed. Among the tested columns, the C18 column with 250 mm length and 4.6 mm diameter demonstrated superior chromatographic performance, yielding two well-defined and adequately separated peaks that complied with ICH system suitability criteria. Guided by previously published studies, various mobile phase compositions and volumetric ratios were explored to enhance the separation of the target analytes. The initial phase of optimization focused on the choice of organic solvent. Methanol and acetonitrile were tested separately, but the mixture of both provided more appropriate retention behavior and improved peak symmetry. Different proportions were subsequently evaluated. Although increasing the acetonitrile content led to reduced retention times, excessively rapid elution was deemed unsuitable for the intended analytical application, particularly for the analysis in the plasma biological matrix. Consequently, the ratio of methanol in the range 35–15% and acetonitrile in the range of 20–5% were evaluated, and 20% methanol and 5% acetonitrile were identified as optimal. The aqueous component of the mobile phase was further modified using various additives, including orthophosphoric acid, potassium dihydrogen phosphate, and an ion-pair agent (sodium octanesulfonate). However, the lack of an ion pair agent in the mobile phase failed to meet acceptable limits for theoretical plate number, resolution, and peak tailing. Subsequent addition of sodium octanesulfonate significantly improved the chromatographic performance. Multiple concentrations were investigated, and the most satisfactory results were achieved at a concentration of 0.25 g/L. Finally, the influence of flow rate and column temperature on method performance was examined. Flow rates ranging from 0.8 to 1.2 mL/min and temperatures between 25 and 40 °C were tested. Optimal assay conditions (Table 1) were established at a flow rate of 1.0 mL/min and a column temperature of 25 °C. Under these conditions, the analytes eluted as sharp, symmetrical peaks with adequate resolution and were monitored at a detection wavelength of 280 nm.

### 2.2. Method Validation

The proposed method was developed and validated using the Analytical Procedure Development guidelines (ICH Q14) [40], Bioanalytical Method Validation (ICH M10) [41] and Bioanalytical Method Validation, Guidance for Industry of the US Department of Health and Human Services Food and Drug Administration [42], and FDA Reviewer Guidance for Validation of Chromatographic Methods of the US Center for Drug Evaluation and Research (CDER) [43].

#### 2.2.1. Selectivity

**Figure 2:**
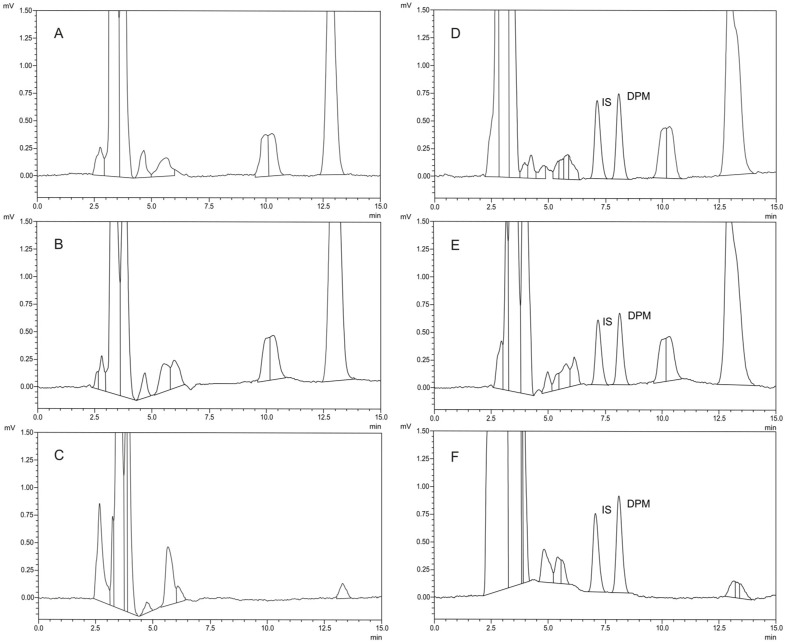
Typical chromatograms obtained from the analysis of blank human plasma (**A**), blank mouse plasma (**B**), blank mouse brain homogenate (**C**), spiked human plasma (calibration standard, 4 μg/mL) (**D**), spiked mouse plasma (calibration standard, 4 μg/mL) (**E**), and spiked mouse brain homogenate (calibration standard, 2 μg/mL) (**F**). The retention times of IS and DPM were 7.07 and 8.03 min, respectively.

Selectivity is measured and evaluated through the analysis of blank biological samples [41,42]. The developed method showed excellent results for DPM determination in different biological media (human/mouse plasma or mouse brain homogenate) using previously described chromatographic conditions. No interferences from the biological matrix were detected. The three representative chromatograms (provided in Figure 2A–C), obtained during the analysis of blank samples, confirmed the specificity and selectivity of the proposed method. It should be noted that DPM is an endogenous compound and, therefore, a truly analyte-free plasma matrix is not available. In the present study, the term “blank plasma” refers to plasma samples that did not exhibit a detectable DPM peak under the validated chromatographic conditions. Since endogenous plasma DPM concentrations reported in the literature are generally in the pg/mL to low ng/mL range [44,45,46], they did not contribute a measurable signal and did not interfere with the quantification of DPM within the validated concentration range.

Although matrix interferences were effectively minimized through the sample preparation procedure, the internal standard (IS) method was applied, and IS was added to correct for analytical variability and ensure robust and accurate quantitation of the target analyte.

#### 2.2.2. Linearity

Linearity was assessed by evaluating the relationship between the analyte’s concentration and the corresponding instrumental response [41,42]. Seven calibration standards, covering the concentration range of 0.5–8.0 µg/mL for human/mouse plasma analysis and 0.25–4.0 µg/mL for mouse brain homogenate assay, were analyzed in triplicate to establish the linearity of the proposed analytical method. Representative chromatograms of several calibration standards were presented in Figure 2D–F.

**Figure 3:**
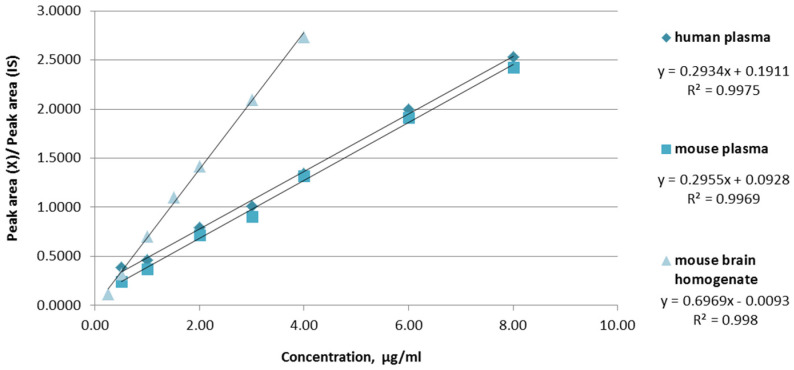
DPM calibration curves in different biological media.

For each biological media, the recovery percentage was determined, and the obtained mean values for DPM were 103.4, 99.9, and 100.5% for human plasma, mouse plasma, and mouse brain homogenate, respectively. All analyzed samples showed adequate accuracy and precision, fulfilling the acceptance criteria specified in the ICH guidelines [41]. Calibration curves were constructed by plotting the area ratio of the analyte’s chromatographic peak to the IS peak against the corresponding analyte concentrations. The linearity of the method across the studied concentration range was confirmed by the regression equations and the high determination coefficients (R^2^) presented in Figure 3.

#### 2.2.3. Accuracy and Precision

In accordance with ICH M10 guidelines [40], the accuracy, precision, and repeatability of the validated analytical method were evaluated using quality control (QC) samples at three concentration levels within the calibration range. In the present study, three QC levels were assessed: low QC (1 μg/mL for the plasma and 0.5 μg/mL for the striatum homogenate assay), medium QC (3 μg/mL for the plasma and 1.5 μg/mL for the striatum homogenate assay), and high QC (6 μg/mL for the plasma and 3 μg/mL for the striatum homogenate assay). For the intraday precision and accuracy, three independently prepared samples at each concentration level were analyzed in triplicate every three hours. Interday performance was evaluated by repeating the same procedure over three consecutive days. Comprehensive data on accuracy, precision, and repeatability are presented in Table 2. Intraday repeatability, expressed as the coefficient of variation (CV%), did not exceed 4.88% for the plasma and 3.32% for the mouse brain homogenate samples across all tested concentration levels. Intraday accuracy, determined as the percentage deviation of the mean measured concentration from the nominal value (d%), ranged from −5.60% to 4.05% for all biological matrices. Interday validation further confirmed the reliability of the method, with CV% values below 10.14% and d% values ranging from −6.82% to 4.56%, as summarized in Table 2.

#### 2.2.4. System Suitability

System suitability tests were performed to verify that the chromatographic system provided adequate performance and resolution for the intended analytical application [41,42,43]. The evaluated parameters depend on the analytical method employed. In the case of the RP-HPLC procedure, the assessed parameters included retention time (t_R_), capacity factor (κ), selectivity (α), number of theoretical plates (N), degree of separation (R_S_), and peak asymmetry (tailing factor, T_f_). Under the optimized chromatographic conditions, the proposed method enabled rapid and efficient separation of the analytes. System suitability was assessed by six replicate injections of the medium QC (3 μg/mL for the plasma and 1.5 μg/mL for the striatum assay). The DPM mean values obtained for the evaluated parameters were κ ≥ 2.5, α ≥ 1.2, N ≥ 3400, R_S_ ≥ 2.8, and T_f_ ≤ 1.4, demonstrating satisfactory system performance. The detailed results are summarized in Table 3.

#### 2.2.5. Limit of Detection (LOD) and Limit of Quantification (LOQ)

The limits of detection and quantification can be determined using either computational or experimental approaches [41,42,43]. In the present study, the experimental method was applied. After serial dilution of the analyte solutions and injection of a 20 μL sample, LOD was established at a signal-to-noise ratio of 2:1, whereas LOQ was determined at a signal-to-noise ratio of 10:1. For the plasma assay, LOD and LOQ were found to be 0.10 μg/mL and 0.50 μg/mL, respectively. Better results were obtained for the mouse brain homogenate assay, with LOD of 0.05 μg/mL and an LOQ of 0.25 μg/mL. The endogenous nature of DPM should be considered when evaluating the applicability of the developed method. Although it is naturally present in plasma, reported physiological concentrations are generally several times lower than the LOQ achieved in the present study [44,45,46]. Therefore, the proposed method is not intended for the direct determination of endogenous plasma DPM at physiological levels. Instead, it is suitable for the quantification of DPM in samples containing concentrations above the validated LOQ, including spiked biological samples, experimental models with elevated DPM levels, and mouse brain homogenates.

#### 2.2.6. Application of the Method

The developed method was used for analysis of DPM in mouse brain homogenate samples from healthy mice and mice receiving fixed doses of 1-methyl-4-phenyl-1,2,3,6-tetrahydropyridine (MPTP) as a part of the acute MPTP-induced mouse model of PD [47]. Ten brain samples (five control samples and five MPTP-treated) were used to determine the target analyte in accordance with the proposed procedure to assess the method’s applicability to actual brain samples. The mean values for the biological samples were 0.27 and 0.19 μg/mL, for the control samples and the MPTP-treated, respectively. The DPM amounts found in mouse brain striatum homogenate are in good agreement with the range of normal values [13,14]. The results of the triple analysis of these MPTP-treated samples showed that the approach is relevant to samples taken from individuals with PD.

### 2.3. Greenness, Whiteness, and Blueness Evaluation

**Figure 4:**
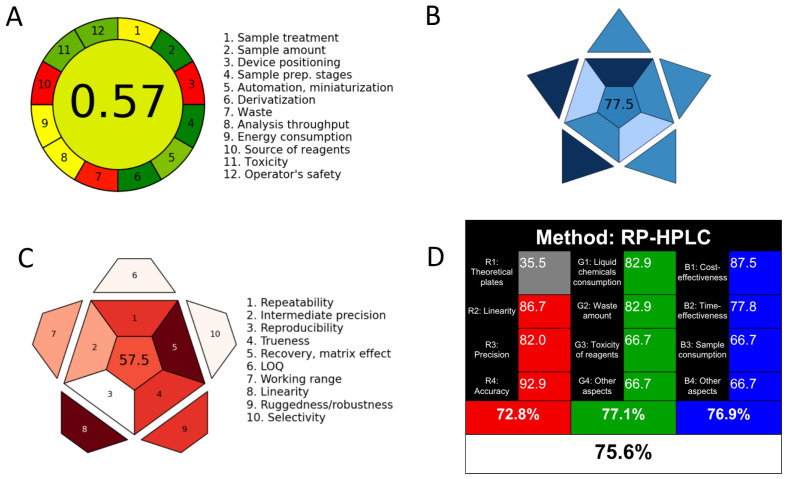
Environmental sustainability evaluation of the proposed RP-HPLC method for determination of DPM and IS; (**A**) AGREE, (**B**) BAGI, (**C**) RAPI, and (**D**) WAC.

The sustainability profile of the developed RP-HPLC method was evaluated using four contemporary analytical assessment metrics—AGREE [36], BAGI [37], RAPI [38], and WAC [39] (Figure 4A–D).

The environmental sustainability of the proposed RP-HPLC method was evaluated using AGREE [36]. Although several greenness assessment tools have been reported in the literature, the AGREE approach is considered among the most comprehensive, as it integrates all 12 principles of Green Analytical Chemistry into a single evaluation framework. The final score ranges from 0.0 to 1.0 and reflects the overall compliance of the method with these principles. The results are presented as a circular pictogram divided into twelve sectors, each corresponding to one of the principles and colored from dark green (1.0) to dark red (0.0). The overall score is displayed at the center of the diagram and is similarly color-coded. According to the AGREE calculator, the developed method achieved a score of 0.57, indicating acceptable environmental performance and suitability for routine analytical application. The AGREE pictogram was shown in Figure 4A, and the AGREE report sheet with AGREE score for each CAC concept was included in Supplementary Figure S1. Since AGREE evaluates the complete set of Green Analytical Chemistry principles with equal consideration, the score of 0.57 suggests that the method can be classified as reasonably green, although further improvements are possible, particularly through the reduction of solvent toxicity and the implementation of more sustainable sample preparation strategies.

The feasibility and practical applicability of the method were evaluated using the BAGI model [37], one of the most recent tools for comprehensive analytical assessment. This model considers ten different parameters describing the analytical procedure and presents the result as an asteroid-shaped pictogram. Each parameter is represented by a specific color scale ranging from white (non-compliance) to dark blue (high compliance), while the overall score is shown at the center of the figure. A value above 60.0 is generally considered indicative of satisfactory method applicability. As illustrated in Figure 4B and Figure S2, the proposed method achieved a BAGI score of 77.5%, confirming the balance between analytical efficiency and environmental burden. This relatively high BAGI value suggests that the developed RP-HPLC procedure successfully combines acceptable analytical performance with reduced environmental impact. These findings support the suitability of the method for routine laboratory applications where sustainability considerations are important.

The analytical performance of the method was additionally assessed using the RAPI, a Python-based open-source tool (v3.14.6) developed for the comprehensive evaluation of quantitative analytical methods [38]. RAPI considers ten key validation-related parameters, including repeatability, intermediate precision, reproducibility, trueness, recovery, matrix effect, limit of quantitation, working range, linearity, ruggedness/robustness, and selectivity. Each criterion is scored on a five-level scale from 0 to 10 and visualized in a star-shaped pictogram using a color gradient ranging from white (lowest performance) to dark red (highest performance). The overall score, calculated as the sum of individual parameter scores (0–100), is displayed at the center of the pictogram and reflects the global analytical performance of the method. The better the overall performance of the method, the higher this value. This graphical approach facilitates rapid interpretation of validation results and supports a comprehensive assessment of method suitability. The RAPI evaluation resulted in a score of 57.5% (Figure 4C and Figure S3), which indicated variability among the evaluated validation and performance parameters. Because RAPI places considerable emphasis on analytical quality and validation performance, the lower value suggests that some method characteristics may still require optimization to achieve superior analytical excellence. Nevertheless, the obtained score remains indicative of a method that satisfies fundamental validation requirements while maintaining a moderate level of analytical sustainability.

The overall sustainability profile of the analytical procedure was further assessed using the WAC model [39], which evaluates method “whiteness” based on three complementary dimensions: analytical performance (red), environmental impact (green), and economic viability (blue). The assessment was conducted using a standardized Excel-based worksheet comprising three sections (red, green, and blue), each scored out of a maximum of 100% based on at least three selected parameters with different impacts. The final table presents the individual color contributions and their combined effect; higher contributions from all three components yield a color closer to white, indicating balanced performance. The proposed method achieved scores of 72.8% (red), 77.1% (green), and 76.9% (blue), resulting in an overall whiteness score of 75.6% (Figure 4D and Figure S4). These results demonstrate a balanced performance across analytical, environmental, and practical dimensions.

A comparison of the four metrics demonstrates that although absolute scores differ, all assessment tools consistently indicate a method with acceptable sustainability characteristics. The AGREE and RAPI scores identify opportunities for further improvement, particularly regarding reagent safety, waste minimization, and selected analytical performance parameters. Conversely, the BAGI and WAC evaluations highlight the method’s strengths in balancing environmental responsibility, analytical reliability, and operational feasibility. The observed variation among the scores is expected because each tool applies different criteria and weighting systems, emphasizing distinct dimensions of analytical sustainability. Overall, the combined assessment confirms that the developed RP-HPLC method possesses a satisfactory sustainability profile and is suitable for routine analytical applications. Further reduction of hazardous solvent use, improvement of chromatographic efficiency, and enhancement of robustness-related parameters may lead to even higher sustainability ratings across all evaluation models.

### 2.4. Study Advantages and Limitations

Notably, several analytical methods for DPM determination have been reported previously. Table 4 presents comparative data for the proposed method and some previously published reports. It can be seen that, in terms of analysis time, linearity, and recovery, the results obtained are better in some cases. However, the novelty of the present work does not reside solely in the chromatographic technique itself, but rather in the combination of simplified sample preparation, reduced analysis time [12,13,18], comprehensive method validation [13,14,22,23], and successful application to different biological samples—human/mouse plasma and mouse brain homogenate [12,13,14,18,19,21,22,23]. While LC-MS/MS methods provide superior sensitivity, the proposed HPLC method offers a cost-effective and widely accessible alternative for routine laboratory analysis. Furthermore, the method requires only 250 µL of biological sample and a simple sample preparation technique—protein precipitation. Further developments should be done in order to extend the proposed method’s application for simultaneous determination of DPM and its metabolites (3,4-dihydroxyphenylacetic acid and homovanillic acid), as well as including the use of a fluorescence detector to improve the method’s sensitivity and precision.

## 3. Materials and Methods

### 3.1. Materials and Reagents

Analytical standards—DPM (4-(2-aminoethyl) benzene-1,2-diol) and 3,4-dihydroxybenzylamine hydrobromide were acquired from Sigma-Aldrich Company, City of St. Louis, MO, USA. The purity of these compounds was more than 98%. Mobile phase and stock solutions were prepared with acetonitrile and methanol of an HPLC grade (with a purity level of more than 99%) purchased from Sigma-Aldrich Company. All required additional chemicals and reagents (potassium dihydrogen phosphate and 1-octanesulfonic acid sodium salt) used to develop the analytical procedure were also of HPLC quality purchased from Sigma-Aldrich Company.

### 3.2. Chromatographic Conditions

A SHIMADZU Corporation system purchased from SHIMADZU Corporation (Kyoto, Japan) was utilized to develop and optimize the recommended technique. This system included a vacuum degasser, pump, auto-injector, and UV-VIS detector. Lab Solution Software v1.25 was used to record and process the collected results. All analyses were conducted using a chromatographic column Avantor ACE^®^ RP-18 (250 × 4.6 mm, 5 µm) equipped with a suitable ODS pre-column. The temperature was ambient, and the mobile phase was composed of 10 mM potassium dihydrogen phosphate buffer (pH = 3) with 0.25 g/L sodium octanesulfonate, methanol, and acetonitrile at a volume-to-volume ratio of 75:20:5. The mixture was filtered through a 0.45 μm membrane filter, and then sonicated for 10 min and pumped in the chromatographic system isocratically at a 1.0 mL/min flow rate. Additionally, the wavelength of the UV detector was set at 280 nm.

### 3.3. Preparation of Stock and Working Solutions

The stock solution of DPM (DPM-SS) was prepared by dissolving 5 mg of the substance in methanol to obtain a 100 µg/mL concentration. Then, two working solutions (D-PL (40 µg/mL) and D-BH (20 µg/mL)) were prepared by pipetting appropriate volumes of the stock solution DPM-SS and diluting with methanol. Finally, seven calibration working solutions (from D-PL1 to D-PL7 for the plasma analysis and from D-BH1 to D-BH7 for the brain homogenate analysis) were prepared and used for the analysis. All the solutions were kept at 2–4 °C.

### 3.4. Preparation of Calibration Standards and Quality Control Samples

Human plasma standard, mouse plasma standard, and mouse brain homogenate from healthy mice were used for the preparation of all calibration and quality control samples. For the analysis, separate aliquots of 400 µL biological matrix (human/mouse plasma or mouse brain homogenate) were mixed with 100 µL of the solutions D-PL1—D-PL7 or D-BH1—D-BH7, and the obtained concentration ranges were 0.5–8 µg/mL and 0.25–4 µg/mL for plasma and brain homogenate analysis, respectively. As a part of the preparation procedure, all blank samples were vortex-mixed for 1 min before adding the calibration working solution and for 2 min after that.

### 3.5. Preparation of Internal Standard Stock and Working Solutions

A molecule with DPM analog structure (3,4-dihydroxybenzylamine hydrobromide, Figure 1) was used as an internal standard (IS). It was dissolved in methanol to prepare a stock solution of 100 µg/mL and further diluted up to 13 or 10 µg/mL for the plasma or brain homogenate analyses, respectively.

### 3.6. Sample Pretreatment

#### 3.6.1. Plasma Samples (Human and Mouse Plasma Standard)

The developed procedure used human plasma standard (Prod. No.: P9523) and mouse plasma standard (Prod. No.: P9275) provided by Sigma-Aldrich Co. Written informed consent and ethics approval for the research were not obtained because the presented method for plasma analysis did not include humans or human tissue samples. To a 150 µL aliquot of plasma, 50 µL of the IS working solution with a concentration of 13 µg/mL was added and vortex-mixed for 1 min. Plasma protein precipitation was made by adding 450 µL acetonitrile. Samples were then vortex mixed for 2 min and centrifuged for 10 min at 13,000 rpm. The supernatant was collected, 600 µL were filtered through a 0.45 µm syringe filter, and 20 µL were injected into the HPLC system for analysis.

#### 3.6.2. Tissue Samples (Mouse Brain, Striatum)

DPM levels were determined in the mouse brain striatum according to a previously developed procedure by our research team [47]. Mice were euthanized, and the striatum was gently dissected, weighed, and stored in a freezer at −80 °C. The procedures were conducted in agreement with the Declaration of Helsinki on the care and use of animals (DHEW publication, NHI 80-23) and the EC Directive 2010/63/EU on animal experimentation. The experimental design was approved by the Bulgarian Food Safety Authority (license number: 418/19.12.2024) on 19 December 2024. Briefly, the biological sample preparation procedure included the following steps: the mixture was homogenized at 3500 rmp/min for 99 s, centrifuged at 12,000 rmp/min for 20 min in a low-temperature high-speed centrifuge, and the supernatant was removed to obtain the biological sample for testing. To a 250 µL aliquot of brain homogenate, 50 µL of the IS working solution with a concentration of 10 µg/mL was added and vortex-mixed for 1 min. Then 200 µL acetonitrile was added, the sample was vortex-mixed for 5 min, and centrifuged for 10 min at 13,000 rpm. The supernatant was collected, 450 µL were filtered through a 0.45 µm syringe filter, and 20 µL were injected into the HPLC system for analysis.

### 3.7. Method Validation Guidelines

According to the International Council for Harmonization (ICH) and US Food and Drug Administration (FDA) guidelines, all analytical and bioanalytical techniques should be validated by the parameters selectivity, linearity, accuracy, precision, system suitability, limit of detection (LOD), and limit of quantification (LOQ). The proposed method was developed using the Analytical Procedure Development guidelines (ICH Q14) [40], and validated using the Bioanalytical Method Validation (ICH M10) [41], Bioanalytical Method Validation, Guidance for Industry of the US Department of Health and Human Services Food and Drug Administration [42], and FDA Reviewer Guidance for Validation of Chromatographic Methods of the US Center for Drug Evaluation and Research (CDER) [43].

## 4. Conclusions

An accurate, reliable, and selective analytical method was developed and validated for the determination of DPM in human or mouse plasma and mouse brain homogenate. The proposed procedure was successfully applied to analyze DPM levels in mouse brain homogenate samples from healthy mice and from mice with an acute MPTP-induced model of PD. AGREE, BAGI, RAPI, and WAC tools clearly demonstrated the sustainability and eco-friendliness of the presented assay and its suitability for implementation in routine analytical practice.

## Figures and Tables

**Table 1:** Optimization of the chromatographic parameters.

Parameter	Tested Conditions	Optimal Conditions
**Stationary** **phase**	C18 and C8 columns with length (150 and 250 mm) and internal diameter (4.0 and 4.6 mm) equipped with suitable ODS pre-columns	Avantor ACE^®^ RP-18 (250 × 4.6 mm, 5 µm) equipped with ODS pre-column (VWR International Ltd., Wien, Austria)
**Mobile phase**	additional reagents: orthophosphoric acid, potassium dihydrogen phosphate, sodium octanesulfonate; ratio of methanol—35–15%; ratio of acetonitrile—20–5%	10 mM potassium dihydrogen phosphate buffer (pH = 3) with 0.25 g/L sodium octanesulfonate: methanol: acetonitrile = 75:20:5
**Flow rate**	0.8–1.2 mL/min	1.0 mL/min
**Wavelength**	230–290 nm	280 nm
**Temperature**	25–40 °C	25 °C

**Table 2:** Intraday and interday precision and accuracy of the developed method (*n* = 3).

Sample Type	DPM Concentration (µg/mL)	Intraday			Interday		
		Mean ± SD	CV%	d%	Mean ± SD	CV%	d%
**Human plasma**	1.0	0.96 ± 0.05	4.88	−3.90	0.99 ± 0.08	8.47	−1.37
	3.0	2.91 ± 0.11	3.72	−3.14	2.80 ± 0.14	4.93	−6.82
	6.0	6.11 ± 0.17	2.81	1.77	6.13 ± 0.06	0.99	2.17
**Mouse plasma**	1.0	0.94 ± 0.01	1.08	−5.60	0.97 ± 0.10	10.14	−3.37
	3.0	2.87 ± 0.07	2.29	−4.28	2.87 ± 0.10	3.42	−4.22
	6.0	6.24 ± 0.18	2.95	4.05	6.27 ± 0.19	3.09	4.56
**Mouse brain homogenate**	0.5	0.49 ± 0.01	1.33	−1.93	0.48 ± 0.02	3.36	−5.07
	1.5	1.55 ± 0.05	3.32	3.56	1.47 ± 0.05	3.42	−1.96
	3.0	2.99 ± 0.06	2.13	−0.38	2.98 ± 0.09	3.01	−0.59

SD: standard deviation; CV: coefficient of variation; d%: percentage deviation of the concentration found from the weighted.

**Table 3:** Results of the system suitability tests (*n* = 6).

Sample Type	Parameter (Acceptance Criteria)	IS	DPM
**Human plasma**	t_R_	7.11 ± 0.02	7.97 ± 0.02
	N (NLT 2000)	3352 ± 43	3462 ± 66
	κ′ (NLT 2.0)	2.24 ± 0.01	2.55 ± 0.01
	α (NLT 1.0)	1.22 ± 0.24	1.25 ± 0.01
	R_S_ (NLT 2.0)	2.44 ± 0.94	2.90 ± 0.03
	T_f_ (NMT 2.0)	1.21 ± 0.07	1.05 ± 0.05
**Mouse plasma**	t_R_	7.08 ± 0.05	8.06 ± 0.05
	N (NLT 2000)	3233 ± 36	3438 ± 58
	κ′ (NLT 2.0)	2.41 ± 0.01	2.74 ± 0.01
	α (NLT 1.0)	1.22 ± 0.07	1.23 ± 0.01
	R_S_ (NLT 2.0)	2.76 ± 0.24	2.85 ± 0.06
	T_f_ (NMT 2.0)	1.29 ± 0.05	0.94 ± 0.05
**Mouse brain homogenate**	t_R_	6.99 ± 0.05	7.99 ± 0.06
	N (NLT 2000)	3669 ± 85	3932 ± 98
	κ′ (NLT 2.0)	2.76 ± 0.02	3.15 ± 0.03
	α (NLT 1.0)	1.51 ± 0.04	1.23 ± 0.01
	R_S_ (NLT 2.0)	2.60 ± 0.76	3.07 ± 0.02
	T_f_ (NMT 2.0)	1.23 ± 0.02	1.33 ± 0.05

t_R_: retention time; N: theoretical plates; κ′: capacity factor; α: selectivity; R_S_: resolution; T_f_: tailing factor; NLT: no less than; NMT: no more than.

**Table 4:** Comparison between the presented HPLC-UV method and the reported chromatographic methods.

Matrix	Method/Detection	t_R_ (min)	Linearity Range (μg/mL)	Recovery (%)	LOD (μg/mL)	LOQ (μg/mL)	Reference (№)
Human/mouse plasma	HPLC-UV	7.0	0.5–8.0	99.9/103.4	0.1	0.5	Proposed method
Mouse brain homogenate	HPLC-UV	7.0	0.25–4.0	100.5	0.05	0.25	Proposed method
Rat urine	HPLC-PDA	4.1	8–42	93.3	1.4	4.23	[12]
Human urine	HPLC-FLD	n.a	0.37–18.9 × 10^−3^	98.3	n.a	0.9 × 10^−4^	[14]
Human urine	UHPLC-MS/MS	3.3	0.005–0.5	n.a	0.3 × 10^−2^	0.1 × 10^−1^	[23]
Mouse brain homogenate	HPLC-FLD	8.5	0.03–2.5	99.3	n.a	0.3 × 10^−1^	[13]
Mouse brain homogenate	HPLC-MS	16.7	0.001–0.7	>90.0	0.1 × 10^−3^	0.1 × 10^−2^	[18]
Brain microdialysates	LC-MS	3.5	0.092–2.298	93.2	0.7 × 10^−1^	0.8 × 10^−1^	[19]
Mice striatum	HPLC-IPAD	n.a	0.08–250 × 10^−4^	102.8	0.2 × 10^−4^	0.8 × 10^−4^	[21]
Brain extracellular fluid	UHPLC-MS	0.9	0.15–16 × 10^−3^	n.a	n.a	0.2 × 10^−3^	[22]

n.a: not available.

## Data Availability

The original contributions presented in this study are included in the article/Supplementary Materials. Further inquiries can be directed to the corresponding author.

## Supplementary Materials

The following supporting information can be downloaded at: https://www.mdpi.com/article/10.3390/molecules31132255/s1, Figure S1: AGREE report sheet with AGREE score for each CAC concept; Figure S2: BAGI report sheet with BAGI score; Figure S3: RAPI report sheet with RAPI score; Figure S4: WAC report sheet with WAC score.

## Abbreviations

The following abbreviations are used in this manuscript:

DPM	Dopamine
PD	Parkinson’s disease
HPLC	High-performance liquid chromatography
RP-HPLC	Reversed-phase high-performance liquid chromatography
UV	Ultraviolet
AGREE	The Analytical Greenness software
WAC	White Analytical Chemistry
BAGI	Blue Applicability Grade Index
IS	Internal standard
ICH	International Council for Harmonization
FDA	US Food and Drug Administration
LOD	Limit of detection
LOQ	Limit of quantification
R^2^	Determination coefficient
QC	Quality control
CV%	Coefficient of variation
d%	Percentage deviation of the concentration found to weighted
t_R_	Retention time
κ	Capacity factor
α	Selectivity
N	Theoretical plates
R_s_	Resolution
T_f_	Tailing factor
SD	Standard deviation
MPTP	1-methyl-4-phenyl-1,2,3,6-tetrahydropyridine
