# Supplementary material for: Bioanalytical HPLC-UV Determination of Dopamine in Plasma and Mouse Brain Homogenate with Greenness, Whiteness, and Blueness Assessment
Source: Molecules. 2026 Jun 26;31(13):2255. doi: 10.3390/molecules31132255 (PMC13362643; doi:10.3390/molecules31132255)
Supplement: Supplementary file 1 [file molecules-31-02255-s001.zip › molecules-4368177-supplementary.pdf]

Analytical Greenness report sheet

16/06/2026 17:51:06

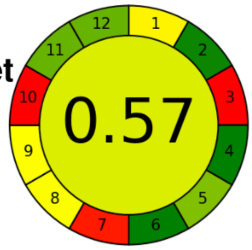

1. Sample treatment
2. Sample amount
3. Device positioning
4. Sample prep. stages
5. Automation, miniaturization
6. Derivatization
7. Waste
8. Analysis throughput
9. Energy consumption
10. Source of reagents
11. Toxicity
12. Operator's safety

Criteria	Score	Weight
1. Direct analytical techniques should be applied to avoid sample treatment.	0.48	2
2. Minimal sample size and minimal number of samples are goals.	0.98	2
3. If possible, measurements should be performed in situ.	0.0	2
4. Integration of analytical processes and operations saves energy and reduces the use of reagents.	1.0	2
5. Automated and miniaturized methods should be selected.	0.75	2
6. Derivatization should be avoided.	1.0	2
7. Generation of a large volume of analytical waste should be avoided, and proper management of analytical waste should be provided.	0.05	2
8. Multi-analyte or multi-parameter methods are preferred versus methods using one analyte at a time.	0.51	2
9. The use of energy should be minimized.	0.5	2
10. Reagents obtained from renewable sources should be preferred.	0.0	2
11. Toxic reagents should be eliminated or replaced.	0.8	2
12. Operator's safety should be increased.	0.8	2

Figure S1. AGREE report sheet with AGREE score for each CAC concept.

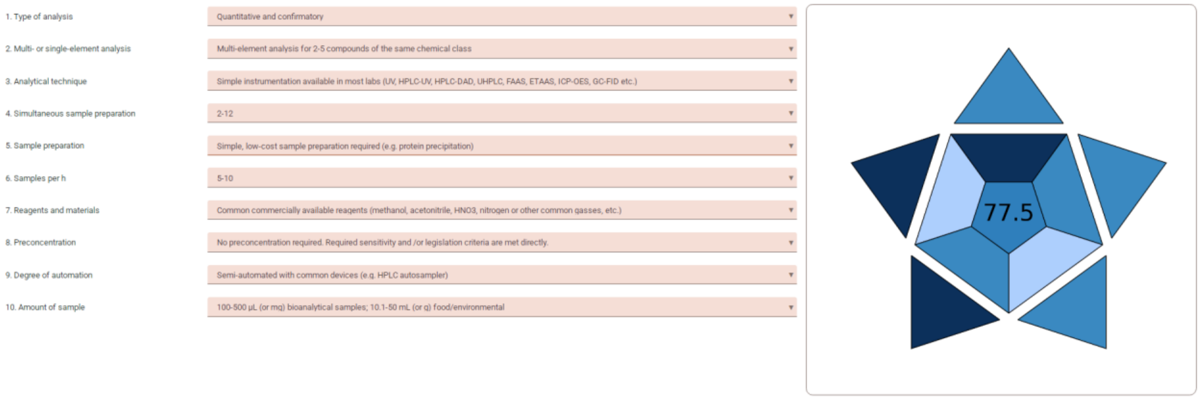

Figure S2. BAGI report sheet with BAGI score.

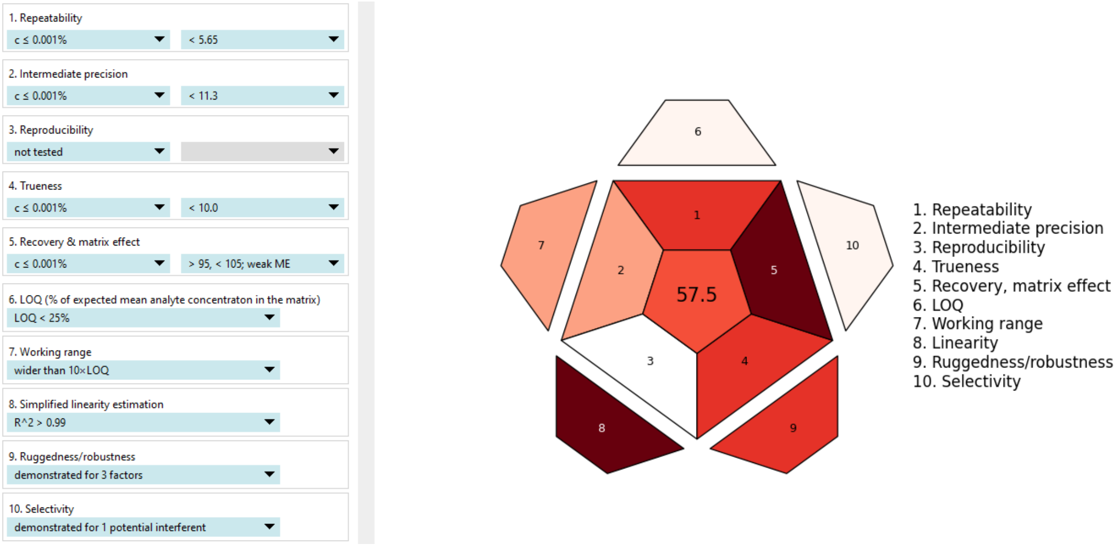

Figure S3. RAPI report sheet with RAPI score.

		w=2		w=2		w=3			w=3		
REDNESS (analytical performance)	W=3	Theoretical plates		Linearity (R2)		Precision (%CV)			Accuracy (%)		
CS: 72.8%	LAV=33.3	2000	2000	0.9900	0.9900	15.00	15.00	15.00	15.00	15.00	15.00
	LSV=66.6	9000	9000	0.9950	0.9950	5.00	5.00	5.00	5.00	5.00	5.00
	Result	3800	3800	0.998	0.998	2.72	2.72	2.72	1.09	1.09	1.09
	Score (0-100)	35.5	35.5	86.7	86.7	82.0	82.0	82.0	92.9	92.9	92.9
		w=3			w=3			w=3			w=1
GREENNESS (safety and eco-friendliness)	W=3	Liquid chemicals consumption (for 10 runs)			Waste amount (for 10 runs)			Toxicity of reagents			Other aspects
CS: 77.1%	LAV=33.3	1000	1000	1000	1000	1000	1000	15	15	15	acceptible
	LSV=66.6	200	200	200	200	200	200	5	5	5	satisfactory
	Result	122	122	122	122	122	122	5	5	5	satisfactory
	Score (0-100)	82.9	82.9	82.9	82.9	82.9	82.9	66.7	66.7	66.7	66.7
		w=3			w=3			w=3			w=1
BLUENESS (productivity / practical effectiveness)	W=3	Cost-effectiveness (USD/ 10 runs)			Time-effectiveness (h/ 10 runs)			Sample consumption (for 10 runs)			Other aspects
CS: 76.9%	LAV=33.3	100	100	100	5	5	5	3.0	3.0	3.0	acceptible
	LSV=66.6	50	50	50	2.5	2.5	2.5	1.5	1.5	1.5	satisfactory
	Result	25	25	25	2	2	2	1.5	1.5	1.5	satisfactory
	Score (0-100)	87.5	87.5	87.5	77.8	77.8	77.8	66.7	66.7	66.7	66.7
FINAL COLOR:		REDNESS		GREENNESS		BLUENESS		BRILLIANCE (MB):		75.6%	
WHITE		≥33.3%	≥66.6%	≥33.3%	≥66.6%	≥33.3%	≥66.6%				
		yes	yes	yes	yes	yes	yes				
Short annotation: 75.6white		Long annotation: 75.6white(72.8/3red-77.1/3green-76.9/3blue)									

Figure S4. WAC report sheet with WAC score.
